# Registry Evaluation of Digital Ulcers in Systemic Sclerosis

**DOI:** 10.1155/2010/363679

**Published:** 2010-08-25

**Authors:** Felice Galluccio, Marco Matucci-Cerinic

**Affiliations:** Division of Rheumatology AOUC, Department of Biomedicine, Denothe Centre, University of Florence, Villa Monna Tessa, viale Pieraccini 18, 50139 Firenze, Italy

## Abstract

Digital ulcers are a very frequent complication of systemic sclerosis affecting about half of the SSc patients, and about 75% of the affected patients have their first DU episode within 5 years from their first non-Raynaud symptom. The lack of adequate classification criteria as well as the lack of knowledge of the development of DU have contributed to the opening of specific registries to better understand the natural history of these lesions. For these reason, specific disease registries play a fundamental role in this field of research. Thanks to the systematic collection of data and their subsequent analysis and comparison between different cohorts, it is possible to improve understanding of the underlying trigger mechanisms of DU development and to determine temporal trends. In the future, the development of recommendations for the management of DU remains of pivotal importance to prevent DU development and obtain rapid healing as well as reduction of pain and disability.

## 1. Introduction

Digital ulcers (DUs) are a very frequent complication of systemic sclerosis (SSc) that affects almost half of the patients, either with limited (lSSc) or diffuse (dSSc) subset of the disease. About 75% of the affected patients have their first DU episode within 5 years from their first non-Raynaud symptom [1–3]. 

The aetiology of DU is complex and multifactorial. The principal mechanisms underlying the DU formation are ischemic, mechanic, and inflammatory, alone or in combination, acting over the SSc vasculopathy [4]. In fact, there are at least three types of DU: those localized in the acral parts of the body, as the fingertips, mainly resulting from an ischemic process, those localized on the dorsal aspect of the fingers where the skin retraction due to fibrosis over bony prominences seems to be the main cause and those evolved on a pitting scar or subcutaneous calcinosis due to a localized inflammatory irritative mechanism [1, 5].

In recent years, the increasing interest for the effects of drugs on healing and prevention of new ulcers and the awareness of the importance of DU for the Quality of life (QoL) of SSc patients, have led to the opening of specific registries to better understand the natural history and evolution of DU.

The present report provides an overview of the DU specific registries that are at the moment available and of the disease registries containing data on DU.

## 2. The Burden of DU

In SSc, DUs are a persistent and often recurrent complication, difficult to manage and slow to heal and can cause tissue loss [6]. Furthermore, DUs are frequently infected and may lead to osteomyelitis, gangrene, autoamputation, and in some cases to septicaemia [7]. 

DUs are also very painful with a disabling effect on patients, limiting hand function and daily activities, such as feeding, dressing, and hygiene [8]. The net effect is the heavy QoL impairment [9]. Indeed, the progressive scarring and tissue loss, that patients experience daily, can lead to severe social and self-esteem problems [10]. Moreover, severe cases of DU required frequent hospitalization with an elevated burden for the health care system and for the families due to the leave from work [8].

## 3. The Scleroderma Digital Ulcers Database (DAS-DU)

The DAS-DU was created in 2004, in the Scleroderma Ulcer Care Unit of the Division of Rheumatology of the University of Florence, to collect information on SSc DU and their management [5]. The DAS-DU includes basic clinical and demographic data that could be useful in clinical trials. Initially, the database was based on a system of paper datasheets that were then stored in chronological order. Despite being a valuable support to understanding the natural history of DU, in practice, this initiative has also identified a number of problems related to opening a long-term database. First, a larger amount of clinical data on DU required a continuous update of the database that was still insufficient to achieve the objective of this initiative. Secondly, the difficulty in data collection and statistical analysis with the paper-based system has led us to create a new electronic database to meet the needs of the progress of knowledge. The database is now maintained on an Access platform stored on a protected offline dedicated computer with anonymised data. All patients are associated with a unique ID, while all DU episodes in a single patient have a secondary unique ID. 

Actually, the DAS-DU represents one of the largest and best characterized DU specific database of a single center SSc cohort. Good quality clinical information on more than 100 SSc patients with more than 2000 DU episodes has been recorded on the database. The dataset collected includes main DU characteristics such as localization (fingertips, nails area, dorsal, and palmar aspect of the finger), dimensions (area in mm^2^), bed of the lesion (reepithelialisation, granulation tissue, fibrin, wet or dry necrosis, eschar, and gangrene), exudate (low, high or pus), borders of the lesions (regular or irregular), perilesional skin (normal or inflamed) and oedema, bone and tendons exposure, and autoamputation. Every DU was also staged in superficial (partial thickness skin loss involving epidermis), intermediate (full thickness skin loss involving damage to or necrosis of subcutaneous tissue that may extend down to, but not through underlying fascia), or deep (full thickness skin loss with extensive destruction or damage to muscle down over the fascia, supporting structures and bone). A pain scale (NRS 0–10) and the Cochin Hand function also known as the Duruöz hand functional disability scale [11, 12] were also included in the main dataset.

Essentially this database reflects the clinical practice in the *Scleroderma Ulcer Care Unit* of our centre which has generated a significant amount of specific consistent data on the main characteristics of DU integrated with clinical features. This has ensured quality and accuracy of the data collected. 

The majority of SSc patients included in the DAS-DU (2004–2008) were limited SSc (70%), most were Caucasian women (86%), mean age of patients was 58 (57.7 ± 15.1 years) with a cumulative mean number of DU of 15.7 ± 17,7 over the four years followup [5]. The majority were observed in lSSc (72%) although the frequency of recurrent DU (61%) were higher in dSSc. DU were most frequently localized on fingertips (55%) with a mean dimension of 50 mm^2^. Mean features of DU were irregular borders of the lesion (80%), and the presence of oedema and inflammation of perilesional skin (75%), and an intermediate stage (60%). Spontaneous moderate to severe pain was always present and is very frequently associated with infection. Despite the treatment, gangrene may occur and osteotendinous exposure (43%) and infection (40%) were frequently observed and required surgical amputation (14%). The database allowed also the record of the time to healing which was about 80 days [5] and also showed that DU, developed on a pre-existing calcinosis, had an increased time to healing to about 95 days and to over 280 days for those derived from gangrene.

## 4. The Digital Ulcers Outcome (DUO) Registry

The DUO Registry, meeting a postapproval commitment to the European Medicines Agency (EMEA), was started in April 2008 to collect information on DU associated with SSc and their management. In this registry, SSc patients with ongoing DU are enrolled to track key information of the clinical course and outcome of DU and to collect safety information on the use of bosentan, a dual endothelin receptor antagonist approved in Europe for reducing the number of new DU in SSc. 

The registry is an European, multicenter, prospective, observational, noninterventional program for patients with DU/SSc. Participating patients undergo assessments and receive medical or surgical therapies according to their physician's judgment. Patients do not receive any experimental intervention or treatment as a result of their participation in the registry. Data are collected via a secure server based on the Internet where the access was guaranteed for registered users only. In order to protect privacy, a unique identifier was assigned to each patient. 

The dataset includes demographics, past history and present status of the disease and DU, complications like soft tissue infection, gangrene or osteomyelitis, and care protocols including topical medications, drugs, and surgical interventions. From April 2008 to May 2009, 648 patients have been enrolled, with 47% lSSc, 41% dSSc, and 9% of overlap/mixed CTD. The mean age was higher for lSSc patient (56.2 years SD:13.4) than dSSc patients (50.8 years SD:14). Preliminary data analysis, still unpublished, shows that more than half of patients needed hospitalization for complications or intervention (59% of lSSc and 52% of dSSc). The most frequent complication observed was soft tissue infection requiring antibiotics (44.5% of lSSc and 40.5% of dSSc), followed by gangrene (34% of lSSc and 26.5% of dSSc) and finally by surgical amputation (17% of lSSc and 11% of dSSc) [13].

## 5. The German Network for Systemic Scleroderma (DNSS) Registry

The DNSS registry was founded in 2003 and includes demographic information of SSc patients and signs and symptoms of organ involvement (heart, lung, gastrointestinal tract, kidney, musculoskeletal system, nervous system, and skin), characteristic laboratory data such as antinuclear antibodies, ESR and CK serum levels, as well as information on physical and on systemic therapies. 

In 2008, a statistical study on more than 1880 SSc patients was performed to detect possible risk factors for the development of DU [14]. About 24% of the patients had active ulcers at the time of entering on the DNSS register. As expected, Raynaud phenomenon (RP) was the most prevalent symptom with a slightly higher prevalence in DU patients (98% versus 94%). Lung fibrosis was significantly higher in patients with DU (45% versus 33%) as well as pulmonary arterial hypertension (PAH: 24% versus 13%), heart (18% versus 13%), upper gastrointestinal (18% versus 13%), and oesophageal involvement (71% versus 57%). Also skin sclerosis (95%) and mouth involvement (36%) were associated with DU. The multivariate analysis of data obtained by comparing patients with or without DU showed that male sex, pulmonary arterial hypertension, oesophageal involvement, the extent of skin sclerosis assessed by the modified Rodnan Skin Score (mRSS), presence of anti-Scl-70 antibodies, young onset of Raynaud's phenomenon (RP), and an elevated ESR represent significant risk factors for the occurrence of DU in SSc. In addition, univariate analysis showed that the above factors and the presence of diffuse subset of disease (dSSc), pulmonary fibrosis and involvement of the upper gastrointestinal tract, and heart involvement are more common in patients with DU representing an elevated relative risk for DU.

Moreover, the combination of male gender, early onset of RP, an ESR >30 mm at the first hour, anti Scl-70 positivity and gastrointestinal and pulmonary arterial involvement showed the highest probability of developing DU (88%) [14].

After all, the DNSS registry showed a heterogeneity between centres for the DU management. In fact, only the 21% of patients with DU received prostacyclins and 40% of patients with DU did not receive a vasoactive therapy, and even in 27.8% of patients who already suffered from DU at the time of initial registration in the DNSS centres, calcium channel blockers was the only therapy. Bosentan (5.5%) and sildenafil (3.4%) are infrequently prescribed in these patients. It was remarkable that 56% of patients with DU performed physiotherapy with paraffin kneading or baths and lymph drainage [15].

## 6. Canadian Scleroderma Research Group (CSRG) Registry

The CSRG have collected annually data on presence, location, and number of DU with their complications and other organ involvement and skin score on their SSc cohort to determine possible associations of DU with other factors such as internal organ complications. 

Out of the 938 patients enrolled, 86% were women with a mean age of 56 years, a disease duration of about 13 years and 53% with lSSc. Fifteen percent patient had currently a DU, 45% had a DU ever, and 53% had pitting scars. Digital necrosis were found on 1.8% of patients and amputation in about 7%. 

A significant association were found with increased disease duration, younger age at the disease onset, Scl-70 autoantibodies, interstitial lung disease, reduced DLCO, increased mRSS, and hands and fingers skin score but no associations with smoking habits, gender, or other structural vasculopathy marker as PAH. It is to be noted that patients with diffuse SSc subset are nearly twice more likely to experience DU than patients with lSSc. Finally, DUs were associated with an increased burden of disease, as assessed by HAQ [16].

## 7. EULAR Scleroderma Trial and Research (EUSTAR) Group

EUSTAR has been founded in 2003 and the minimal essential data set (MEDS) has been created to allow all the members to store their data. In the first analysis of the database, which included a total of 3656 patients (1349 with dSSc and 2101 with lSSc) enrolled in over than 100 centres worldwide, DU resulted to be in 42.7% of patients with dSSc and about 33% of lSSc patients. In both subsets, patient with earlier onset of Raynaud's phenomenon had DU more often than those with a late onset (51% versus 35% in dSSc and 39% versus 28% in lSSc) [2]. Moreover, a difference of prevalence of DU in association with autoantibodies was demonstrated. Indeed, the prevalence was 36.7% with ANA positive, 44.8% with Scl70 positive, and 31.2% with ACA positive [2]. This has led EUSTAR to start a DU observational study with a retrospective and a prospective phase, opened to all member centres, with the aim to describe the natural history, treatment patterns, and outcomes of DU disease in a multinational cohort of incident DU patients. Actually, more than 120 patients have already been enrolled in retrospective phase and about 60 in perspective. Preliminary data will be available soon. Single center and national registries, as above mentioned, are very useful to understand the prevalence and incidence of DU in SSc but an international registry remains of invaluable utility to understand the overall behaviour of DU in SSc and its clinical features as well as being a solid base for large scale clinical and basic studies.

## 8. Assessment and Outcome Measure

The definition of outcome measure and indicators of DU severity is perhaps the main purpose of the DU registries. In recent years, several trials have studied the effects of drugs on healing or prevention of DU [10, 17–20] but, analyzing these studies, it is clear that DUs are *differently defined* and there is disagreement on the *outcome measures* used. 

At the best of our knowledge, there are a considerable number of possible markers of DU *severity*: dimensions, depth, localization, origin, loss of tissue with bone and tendon exposure, inflammation or infection of perilesional tissues, pain, and loss of hand function [5]. A large and deep DU is associated with longer time to healing as well as the bone exposure might be associated with osteomyelitis that may require surgical amputation [5]. Loss of hand function, hand disability, and QoL may also become markers of DU severity because they may have a substantial impact on patient well-being [9]. 

*Localization *may be helpful in determining the main trigger of DU. Usually, DU over the finger joints have been considered mechanical, mainly due to skin retraction and independent from ischemia [1] as the dorsal microvascular perfusion seems maintained in these area while the microvascular alterations are concentrated on the fingertips [21]. 

*Pain* is a pivotal symptom because it seems associated with severity of DU. Spontaneous moderate to severe pain is almost always present in large and deep DU, probably due to the extensive ischemia in the underlying tissues, but the presence of severe pain at DU presentation or the worsening of pain may be linked, in the majority of cases, with infection. Thus, the cause of pain must be carefully investigated in daily practice [5].

The past history of DU or DU as early manifestation of SSc may be a predictor of the future course and may help physicians in selecting a preventive aggressive treatment.

## 9. Conclusion

In SSc, a clinical classification of DU is still lacking, and today the treatment and prevention of DU represent a stimulating challenge for all rheumatologists. 

The increasing number of clinical trials on DU highlights the need to increase knowledge of the mechanisms underlying the formation of DU in SSc. This has been the main reason for the creation of specific DU registries that have now a fundamental role in this field of research. 

Thanks to the systematic collection of data and their subsequent analysis and comparison between different cohorts, it is possible to improve understanding of the different aspects of DU development and to determine therapeutic temporal trends [22–24].

The weak points of these registries, however, remain the lack of unambiguous criteria for the assessment of DU that are potential for selection bias, as well as the lack of data verification and probably patients are not monitored as rigorously as in randomized controlled studies (RCTs) thus leading to underestimation of the rate of some “events”. Moreover, the lack of internationally accepted outcome measure has led to nonuniformity of the published. This evidence suggests to the community the urgent need to design a common strategy on DU mainly based on scientific evidence. Moreover, the DU classification still remains an unmet need that indeed may be helpful in identifying those lesions to be included or excluded from clinical trials [25]. The discrepancy of results in efficacy of some drugs seen in the trials published until today, may be probably due to the multifactorial pathogenesis of DU. In fact, DU caused by a predominantly ischemic process may be more responsive to vasoactive therapy than those caused by skin retraction or calcinosis.

There are some differences between disease registries and registries created for a specific feature of the disease. Data from a specific registry are more accurate than those derived from a general disease registry. In fact, some “event” might be underestimated, probably due to the amount of data included to the aim of the registry which is often to verify the natural course of disease and response to therapies. For this reason, it is fundamental to create specific registries, like EUSTAR and DUO registry, whose sole purpose is to study ongoing DU in SSc.

In the future, the development of recommendations for the management of DU remains of pivotal importance to obtain rapid healing as well as reduction of pain and disability.
